# Stroke Risk Prediction with Machine Learning Techniques

**DOI:** 10.3390/s22134670

**Published:** 2022-06-21

**Authors:** Elias Dritsas, Maria Trigka

**Affiliations:** Department of Computer Engineering and Informatics, University of Patras, 26504 Patras, Greece; trigka@ceid.upatras.gr

**Keywords:** stroke, risk prediction, machine learning, data analysis

## Abstract

A stroke is caused when blood flow to a part of the brain is stopped abruptly. Without the blood supply, the brain cells gradually die, and disability occurs depending on the area of the brain affected. Early recognition of symptoms can significantly carry valuable information for the prediction of stroke and promoting a healthy life. In this research work, with the aid of machine learning (ML), several models are developed and evaluated to design a robust framework for the long-term risk prediction of stroke occurrence. The main contribution of this study is a stacking method that achieves a high performance that is validated by various metrics, such as AUC, precision, recall, F-measure and accuracy. The experiment results showed that the stacking classification outperforms the other methods, with an AUC of 98.9%, F-measure, precision and recall of 97.4% and an accuracy of 98%.

## 1. Introduction

According to the World Stroke Organization [1], 13 million people get a stroke each year, and approximately 5.5 million people will die as a result. It is the leading cause of death and disability worldwide, and that is why its imprint is serious in all aspects of life. Stroke not only affects the patient but also affects the patient’s social environment, family and workplace. In addition, contrary to popular belief, it can happen to anyone, at any age, regardless of gender or physical condition [2].

A stroke is defined as an acute neurological disorder of the blood vessels in the brain that occurs when the blood supply to an area of the brain stops and the brain cells are deprived of the necessary oxygen. Stroke is divided into ischemic and hemorrhagic. It can be mild to very severe with permanent or temporary damage. Hemorrhages are rare and involve the rupture of a blood vessel resulting in cerebral hemorrhage. Ischemic strokes, which are the most common, involve the cessation of blood flow to an area of the brain due to a narrowing or blockage of an artery [3,4].

Factors that increase the chance of having a stroke are the existence of a similar stroke in the past, the existence of a transient stroke, the presence of myocardial infarction, and other heart diseases, such as heart failure, atrial fibrillation, age (if someone is over 55 years of age, they are clearly more likely to be affected, although stroke is described at any age, even in children), hypertension, carotid stenosis from atherosclerosis, smoking, high blood cholesterol, diabetes, obesity, sedentary lifestyle, alcohol consumption, blood clotting disorders, estrogen therapy and the use of euphoric substances such as cocaine and amphetamines [5,6,7].

Moreover, stroke progresses rapidly, and its symptoms can vary. Symptoms can sometimes develop slowly and sometimes it can develop quickly. It is even possible for someone to wake up while sleeping with symptoms. A stroke occurs with the sudden onset of one or more symptoms. The main ones are paralysis of the arms or legs (usually on one side of the body), numbness in the arms or legs or sometimes on the face, difficulty speaking, difficulty walking, dizziness, decreased vision, headache and vomiting and a drop in the angle of the mouth (crooked mouth). Finally, in severe strokes, the patient loses consciousness and falls into a coma [8,9].

Once the patient has had a stroke, a computerized tomography (CT) scan immediately provides a diagnosis. In the case of ischemic stroke, magnetic resonance imaging (MRI) is efficient. Other ancillary diagnostic tests are carotid triplex and cardiac triplex. Strokes can be severe (extensive) or mild. In the vast majority of cases, the first 24 h are crucial. The diagnosis will highlight the treatment, which is usually pharmaceutical, and, in a few cases, surgical. Intubation and mechanical ventilation in the intensive care unit are necessary when the patient has fallen into a coma [10,11].

Although some patients recover after a stroke, the vast majority continue to have problems depending on the severity of the stroke, such as memory, concentration and attention problems, difficulty speaking or understanding speech, emotional problems such as depression, loss of balance or the ability to walk, loss of sensation on one side of the body and difficulty swallowing food [12,13].

Recovery helps to regain lost function after a stroke. The appropriate plan is created so that the patient immediately returns psychologically and socially with kinesiotherapy, speech therapy and the contribution of neurologists [14,15]. In order to minimize the chances of having a stroke, it is necessary to regularly monitor blood pressure, exercise regularly, maintain a normal weight, quit smoking and drinking alcohol and follow a healthy diet without fat and salt [16,17].

Information and communication technologies (ICTs), and especially the fields of artificial intelligence (AI) and machine learning (ML), now play an important role in the early prediction of various diseases, such as diabetes (as a classification [18] or regression task for continuous glucose prediction [19,20]), hypertension [21], cholesterol [22], COVID-19 [23], COPD [24], CVDs [25], ALF [26], sleep disorders [27], hepatitis C [28], CKD [29], etc. In particular, the stroke will concern us in the context of this study. For this specific disease, many research studies have been conducted with the aid of machine learning models.

In this research work, a methodology for designing effective binary classification ML models for stroke occurrence is presented. Since class balancing is crucial for the design of efficient methods in stroke prediction, the synthetic minority over-sampling technique (SMOTE) [30] method was applied. Then, various models are developed, configured and assessed in the balanced dataset. For our purpose, naive Bayes, logistic regression, stochastic gradient descent (SGD), K-NN, decision trees, random forests and multi-layer perception were evaluated. In addition, the majority voting and stacking methods were applied, with the latter being the main contribution of the current study. The experiments revealed the efficacy of the stacking method against the single models and the voting, achieving a high AUC, precision, recall, F-measure and accuracy.

The rest of the paper is organized as follows. Section 2 describes the relevant works with the subject under consideration. Then, in Section 3, a dataset description and analysis of the methodology followed is made. In addition, in Section 4, we describe the experimental setup and discuss the acquired research results. Finally, conclusions and future directions are outlined in Section 5.

## 2. Related Work

The research community has shown great interest in developing tools and methods for monitoring and predicting various diseases that have a significant impact on human health. In this section, we will present the latest works that utilize machine learning techniques for stroke risk prediction.

Firstly, the authors in [31] applied four machine learning algorithms, such as naive Bayes, J48, K-nearest neighbor and random forest, in order to detect accurately a stroke. The accuracy of the naive Bayes classifier was 85.6%, whereas the accuracy for J48, K-nearest neighbor and random forest was 99.8%.

In [32], the authors proposed a methodology in order to find out the various symptoms associated with the stroke disease and preventive measures for a stroke from social media resources. They defined an architecture for clustering tweets based on the content iteratively using spectral clustering. For the experiments, the ten-fold cross-validation, naive Bayes, support vector machine and probability neural network (PNN) were applied. The PNN had a better performance compared to other algorithms, with an accuracy of 89.90%.

In addition, in [33], logistic regression, naive Bayes, Bayesian network, decision tree, neural network, random forest, bagged decision tree, voting and boosting model with decision trees were applied in order to classify stroke risk levels. The experiment results showed that the boosting model with decision trees achieved the highest recall (99.94%), whereas the random forest achieved the highest precision (97.33%).

Moreover, the Kaggle dataset [34] is applied in [35]. This research work suggests the implementation of various machine learning algorithms, such as logistic regression, decision tree, random forest, K-nearest neighbor, support vector machine and naive Bayes. The naive Bayes, compared to the other algorithms, achieved a better accuracy, with 82% for the prediction of stroke.

In addition, the authors in [36] aim to acquire a stroke dataset from Sugam Multispecialty Hospital, India and classify the type of stroke by using mining and machine learning algorithms. The categories of support vector machine and ensemble (bagged) provided 91% accuracy, while an artificial neural network trained with the stochastic gradient descent algorithm outperformed other algorithms, with a higher classification accuracy greater than 95%.

In addition, an analysis of patients’ electronic health records in order to identify the impact of risk factors on stroke prediction was performed in [37]. The classification accuracy of the neural network, decision tree and random forest over 1000 experiments on the dataset of electronic health records was 75.02%, 74.31% and 74.53%, respectively.

Finally, in [38], the ability of ML techniques to analyze diffusion-weighted imaging (DWI) and fluid-attenuated inversion recovery (FLAIR) images of patients with stroke within 24 h of symptom onset was investigated by applying automatic image processing approaches. Three ML models were developed to estimate the stroke onset for binary classification (≤4.5 h), such as logistic regression, support vector machine and random forest. The ML model evaluation was based on the sensitivity and specificity for identifying patients within 4.5 h and compared to the ones of human readings of DWI-FLAIR mismatch.

## 3. Materials and Methods

### 3.1. Dataset Description

Our research was based on a dataset from Kaggle [34]. From this dataset, we focused on participants who are over 18 years old. The number of participants was 3254, and all of the attributes (10 as input to ML models and 1 for target class) are described as follows:

- **Age** (years) [39]: This feature refers to the age of the participants who are over 18 years old.
- **Gender** [39]: This feature refers to the participant’s gender. The number of men is 1260, whereas the number of women is 1994.
- **Hypertension** [40]: This feature refers to whether this participant is hypertensive or not. The percentage of participants who have hypertension is 12.54%.
- **Heart_disease** [41]: This feature refers to whether this participant suffers from heart disease or not. The percentage of participants suffering from heart disease is 6.33%.
- **Ever married** [42]: This feature represents the marital status of the participants, 79.84% of whom are married.
- **Work type** [43]: This feature represents the participant’s work status and has 4 categories (private 65.02%, self-employed 19.21%, govt_job 15.67% and never_worked 0.1%).
- **Residence type** [44]: This feature represents the participant’s living status and has 2 categories (urban 51.14%, rural 48.86%).
- **Avg glucose level** (mg/dL) [45]: This feature captures the participant’s average glucose level.
- **BMI** (Kg/m^2^) [46]: This feature captures the body mass index of the participants.
- **Smoking Status** [47]: This feature captures the participant’s smoking status and has 3 categories (smoke 22.37%, never smoked 52.64% and formerly smoked 24.99%).
- **Stroke**: This feature represents if the participant previously had a stroke or not. The percentage of participants who have suffered a stroke is 5.53%.

Most features are nominal except for the age, average glucose level and BMI, which are numerical.

### 3.2. Long-Term Stroke Risk Assessment

To assess the long-term risk of stroke occurring, the initial dataset was separated into a training and a test set. A binary variable *c* denotes the class label of an instance *i* in the dataset. The class variable has two possible states, e.g., $c=‘‘Stroke^{\prime \prime }$ or $c=‘‘Non-Stroke^{\prime \prime }$. The risk factors associated with stroke constitute the features with which ML models are fed to predict the class of new instance. The features vector of an instance *i* is denoted as $f_{i}=\left(\begin{array}{c} f_{i1},f_{i2},\cdots ,f_{in} \end{array}\right)$.

The following analysis aims to design machine learning models that achieve high recall (or, else, sensitivity) and area under curve, ensuring the correct prediction of stroke instances. The proposed methodology for stroke prediction consisted of several steps, which are explained below.

#### Data Preprocessing

The raw data quality may degrade the final prediction quality, either due to missing values and/or noisy data. Hence, data preprocessing is necessary, including redundant values reduction, feature selection and data discretization to make it more appropriate for mining and analysis [48]. In addition, part of data preprocessing is class balancing via the employment of a resampling method. In the proposed framework, we employed the so-called SMOTE [30] to address the imbalanced distribution of participants among the stroke and non-stroke classes. More specifically, the minority class, in this case, the ‘stroke’, was oversampled, such that the participants were equally distributed. In addition, there were not missing or null values, so neither dropping nor data imputation was applied.

**Figure 1:**
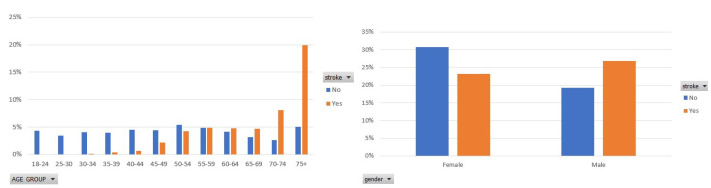
Participants distribution per age group and gender type in the balanced dataset.

Figure 1 illustrates the participants’ distribution in each class in terms of the age group that they belong to and the gender of each participant. Focusing on the stroke class, in the left figure, a significant percentage of the participants are older than 74 years, whereas the second, most frequently occurring age group is 70–74. In addition, in this figure, we see that stroke mainly concerns elderly people. In the right figure of Figure 1, the percentage of women and men who had a stroke is approximately 23% and 26%, respectively. That shows that men are by 3% more prone to stroke disease, which, however, still targets men and women.

**Figure 2:**
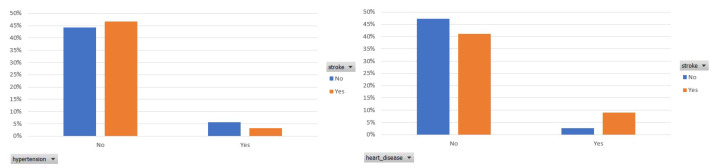
Participants distribution per hypertension and heart disease status in the balanced dataset.

In the following, Figure 2 presents the prevalence of hypertension and heart disease among the participants who had a stroke. In both figures, we observe that an essential ratio of participants who had a stroke has not been diagnosed with hypertension or heart disease.

**Figure 3:**
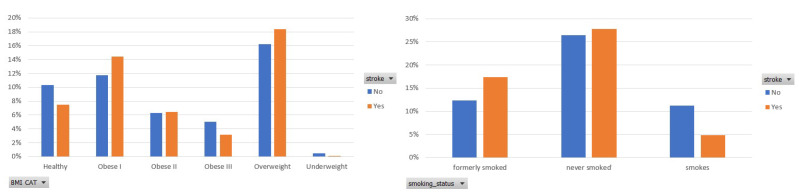
Participants distribution per BMI category and smoke status in the balanced dataset.

Next, Figure 3 depicts the participants’ distribution among the six categories of BMI [19] and the three ones of smoking habits. As for the BMI, an important number of participants (25%) belong to an obese class, whereas 18% of them are overweight. The importance of BMI is also captured by the ranking score assigned by the selected feature importance method in the balanced data.

**Figure 4:**
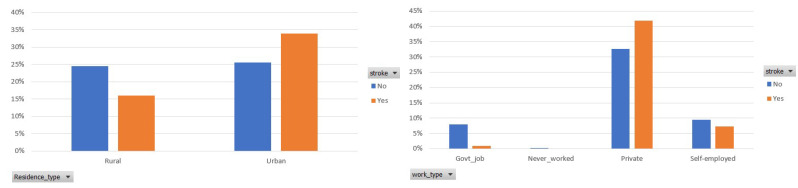
Participants distribution per residence and work type in the balanced dataset.

Finally, Figure 4 demonstrates the participants’ distribution among the two classes, in terms of the residence and work type. It is observed that 34% of the participants who had a stroke live in the urban area, whereas 16% of them live in the rural area. In addition, most of the participants’ (75%) occupation is private and 42% of them had a stroke.

In classification analysis, feature importance constitutes a core component that facilitates the development of accurate and high-fidelity ML models. The accuracy of the classifiers improves until an optimal number of features is considered. The performance of ML models may deteriorate if irrelevant features are assumed for the models’ training. Feature ranking is defined as the process of assigning a score to each feature in a dataset. In this way, the most significant or relevant ones are considered, namely, those ones that may contribute greatly to the target variable to enhance the model accuracy.

In Table 1, we present the dataset features’ importance concerning the stroke class. For this purpose, we considered two different methods. The former utilize a random forest classifier to assign a ranking score, whereas the latter is based on the information gain method [49]. Both methods show that the age is the most important and relevant risk factor for the occurrence of stroke. In addition, we observe that each method has assigned a different ranking order for the rest features, except for the work type and hypertension. The feature hypertension is last in the ordering because, in the dataset, a significant percentage of participants who have had a stroke do not suffer from hypertension. Moreover, all scores are positive, which means that the features may enhance the models’ performance.

### 3.3. Machine Learning Models

In this section, we present the models that will be utilized in the classification framework for stroke occurrence. For this purpose, various types of classifiers are employed.

#### 3.3.1. Naive Bayes

Firstly, the naive Bayes (NB) classifier was considered, which ensures probability maximization if the features are highly independent [50]. A new subject *i* with features vector $f_{i}$ is classified at that class *c* for which $P\left(c\right|f_{i1},\ldots ,f_{in})$ is maximized. The conditional probability is defined as

(1) $$P\left(c\right|f_{i1},\ldots ,f_{in})=\frac{P\left(f_{i1},\ldots ,f_{in}|c\right)P\left(c\right)}{P\left(f_{i1},\ldots ,f_{in}\right)}$$

where $P\left(f_{i1},\ldots ,f_{in}|c\right)=\prod_{j=1}^{n}P\left(f_{ij}|c\right)$ is the features probability given class, $P\left(f_{i1},\ldots ,f_{in}\right)$ is the prior probability of features and P(c) is the prior probability of class. The maximization of (1) was achieved by maximizing its numerator, formulating the following optimization problem

(2) $$\hat{c}=\arg\max P\left(c\right)\prod_{j=1}^{n}P\left(f_{ij}|c\right),$$

where c∈{Stroke,Non−Stroke}.

#### 3.3.2. Random Forest

Random forest (RF) [19] ensembles many independent decision trees and, by resampling, creates different subsets of instances to perform classification and regression tasks. Each decision tree exports its own classification outcome, and then the final class is derived through majority voting.

#### 3.3.3. Logistic Regression

Another model, which will be part of the proposed framework, is logistic regression (LR) [51]. It is a statistical classification method, initially designed for binary tasks that have been extended to multi-class ones as well. The model output is a binary variable in which p=P(Y=1) denotes the probability of an instance to belong in the “Stroke” class, thus 1−p=P(Y=0) captures the probability of an instance belonging in the “Non-Stroke” class. The linear relationship between log-odds with base *b* and model parameters $\beta_{i}$ is as follows:

(3) $$log_{b}\left(\frac{p}{1-p}\right)=\beta_{0}+\beta_{1}f_{i1}+\cdots +\beta_{n}f_{in}$$

#### 3.3.4. K-Nearest Neighbors

K-nearest neighbors (K-NNs) classifier is a distance-based (i.e., Euclidean, Manhattan) method that computes similarity or difference between two instances in the dataset under investigation [52]. The Euclidean distance is the simplest and most commonly used. Let $f_{new}$ be the features vector of the new sample to be classified either as stroke or non-stroke. The KNN classifier determines the closest *K* vectors (neighbors) to $f_{new}$. Then, $f_{new}$ is assigned to the class that most of its neighbors belong to.

#### 3.3.5. Stochastic Gradient Descent

Stochastic gradient descent (SGD) [53] is an optimization technique that can be utilized to learn various linear models and does not belong to a specific family of ML models. It is an efficient approach that, at each iteration, computes the gradient using a single sample. It allows minibatch, and thus it is suitable for large-scale problems.

#### 3.3.6. Decision Tree

For the development of decision tree (DT) [54], we considered J48 as single classifier and RepTree [55] as base classifier in the stacking method. The internal nodes of a DT represent a feature, and the leaf nodes denote the classes. J48 splits a single feature at each node using the Gini index, whereas the latter is a simple and fast decision learner that builds a decision tree using information gain as an impurity measure and prunes it using reduced-error pruning.

#### 3.3.7. Multilayer Percepton

A multilayer perceptron (MLP) is a fully connected feedforward artificial neural network (ANN). The neurons in the MLP are trained with the backpropagation learning algorithm. MLPs are designed to approximate any continuous function and can solve problems that are not linearly separable [56,57].

#### 3.3.8. Majority Voting

Assuming an ensemble of *K* basis models, simple majority voting applies hard or soft voting to predict the class label of an input instance [58]. The former aggregates the votes that relate to each class label and outputs the one with the most votes as a candidate class. The latter sums the predicted probabilities for each class label and predicts the class label with the largest probability. Here, hard voting was adopted. Its general function is captured by the following equation:

(4) $$\max\sum_{k=1}^{K}P_{k,c},$$

where $P_{k,c}$ is the prediction or probability of *k*-th model in class *c*, where c={Stroke,Non−Stroke}.

#### 3.3.9. Stacking

Stacking [59] belongs to ensemble learning methods that exploit several heterogeneous classifiers whose predictions were, in the following, combined in a meta-classifier. The base models were trained on the training set, whereas the meta-model was trained on the outputs of the base models. In this study, the stacking ensemble comprises naive Bayes, random forests, RepTree [54] and J48 [60] as base classifiers, whose predictions were used to train a logistic regression meta-classifier.

### 3.4. Evaluation Metrics

Under the evaluation process of the considered ML models, several performance metrics were recorded. In the current analysis, we will consider the most commonly used in the relevant literature [61].

Recall (true positive rate) or, otherwise, sensitivity, corresponds to the proportion of participants who had a stroke and were correctly considered as positive, with respect to all positive participants. Precision and recall are more suitable to identify the errors of a model when dealing with imbalanced data. Precision indicates how many of those who had a stroke actually belong to this class. Recall shows how many of those who had a stroke are correctly predicted. *F-measure* is the harmonic mean of the precision and recall and sums up the predictive performance of a model.

(5) $$\begin{array}{ccccc} Recall=\frac{TP}{TP+FN}, & \; & Precision=\frac{TP}{TP+FP} & \; & \end{array}$$

(6) $$\begin{array}{ccc} F-\mathrm{Measure}=2\frac{Precision\cdot Recall}{Precision+Recall}, & \; & Accuracy=\frac{TN+TP}{TN+TP+FN+FP} \end{array}$$

Notice that *TP*: true positive, *TN*: true negative, *FP*: false positive and *FN*: false negative.

Area under curve (AUC) is a useful metric, whose values lie in the range [0,1]. The closer to one, the better the ML model performance is in distinguishing stroke from non-stroke instances. The perfect discrimination among the instances of two classes means that the AUC equals one. On the other side, when all non-strokes are classified as strokes and vice versa, the AUC equals 0.

## 4. Results and Discussion

### 4.1. Experiments Setup

In this section, the ML models performance is evaluated in the WEKA 3.8.6 [62] environment. WEKA is a free JAVA-based data mining tool created and distributed under the GNU General Public License. It provides a library of various models for data preprocessing, classification, clustering, forecasting, visualization, etc. The PC in which experiments were carried out has the following characteristics: Intel(R) Core(TM) i7-9750H CPU @ 2.60 GHz 2.59 GHz 16 GB Memory, Windows 10 Home, 64-bit Operating System, x64-based processor. For our experiments, 10-cross validation was applied to assess the models’ efficiency in the balanced dataset of 6148 instances.

For the implementation of the stacking model, four base classifiers were combined. More specifically, naive Bayes, random forest, J48 and RepTree were selected, and their outcomes were fed into a logistic regression meta-classifier. As for the majority voting, we considered the same models with the stacking method, except for naive Bayes. J48 was used to design the decision tree model. In the Weka tool, J48 is an open-source implementation of the C4.5 algorithm. The settings of J48 were as follows: the confidence factor was set to 0.25, and unpruned was set to false. The minimum number of instances per leaf node was set to the default value, and the binary split was set to false. Concerning the MLP, the hidden layers were configured to ‘a’, the learning rate was set to 0.3, the momentum factor was 0.2 and the training time was 500. The momentum term involves weight updates and attempts to improve the convergence speed and avoid stacking at local minima [63].

### 4.2. Evaluation

**Figure 5:**
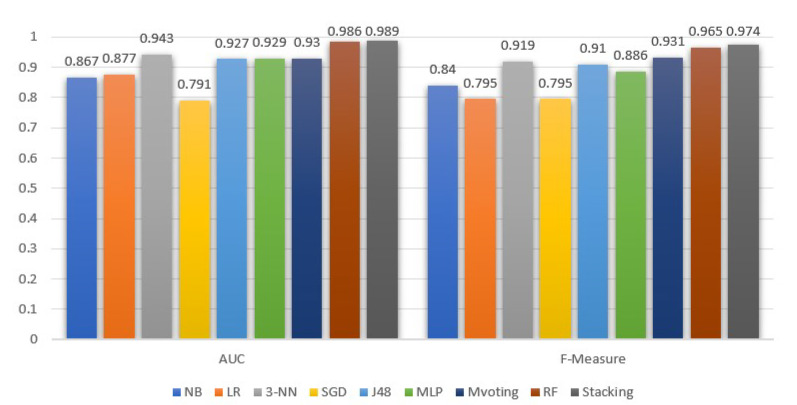
Machine learning models AUC and F-measure evaluation for the stroke class.

**Figure 6:**
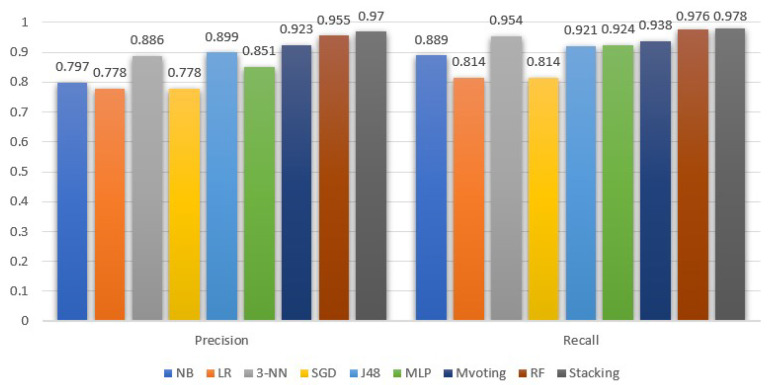
Machine learning models precision and recall evaluation for the stroke class.

Figure 5 and Figure 6 demonstrate the ML models performance, exclusively for the stroke class, in terms of precision, recall, F-measure and AUC. In addition, in Table 2, we summarize the average performance of the selected models.

The stacking model under the selected base models was the most efficient in all metrics under consideration. Similarly, high values were achieved by the RF and majority voting classifiers. Focusing on the AUC metric, the stacking and RF models have approximately similar discrimination abilities, which show that, with a high probability of 98.9% and 98.6%, respectively, both models can successfully identify the stroke from the non-stroke instances. Besides stacking and RF, the 3-NN model is the next one, with an essentially high AUC equal to 94.3%.

Moreover, comparing Figure 5 and Table 2, we observe that the AUC values for the stroke class follow the average behavior. Contrary to AUC, the precision metric for the stroke class is higher than the average. As for the recall metric, the outcomes for the stroke class are higher than the average performance for all models, except for RF, which is 0.1% lower compared to its mean performance. In addition, from Figure 6, we see that, in the case of stroke class, the recall metric values are more or less higher than the precision metric. Notice that the stacking classifier performed a better recall than the rest.

In addition, comparing the average precision and recall, they are either equal, or the former is 0.2–0.3% higher than the latter. A higher difference with a precision lower than the recall is observed in the naive Bayes model. In either case, since the dataset is balanced, the F-measure is a suitable ratio that can reflect the performance (i.e., the accuracy) of the ML models on the dataset. From the F-measure perspective, stacking is 0.8% higher than the RFs, and 5.9% and 6.5% higher than 3-NN and DT (J48), respectively.

In Table 3, the outcomes of the current research work are compared with the research study in [35] under the same dataset [34]. In relation to [35], all suggested models, especially DT and RF, significantly outperform their performance, in terms of recall, F-measure and accuracy. In conclusion, the stacking method remains the best performing method and the main suggestion of our study.

A limitation of this study is that it was based on a publicly available dataset. These data are of specific size and features as opposed to data from a hospital or institute. Although the latter could give more rich information data models with various features capturing a detailed health profile of the participants, acquiring access to such data is usually time-consuming and difficult for privacy reasons.

## 5. Conclusions

A stroke constitutes a threat to a human’s life that should be prevented and/or treated to avoid unexpected complications. Nowadays, with the rapid evolution of AI/ML, the clinical providers, medical experts and decision-makers can exploit the established models to discover the most relevant features (or, else, risk factors) for the stroke occurrence, and can assess the respective probability or risk.

In this direction, machine learning can aid in the early prediction of stroke and mitigate the severe consequences. This study investigates the effectiveness of various ML algorithms to identify the most accurate algorithm for predicting stroke based on several features that capture the participants’ profiles.

The performance evaluation of the classifiers using AUC, F-measure (which summarizes precision and recall) and accuracy is essentially suitable for the models’ interpretation, demonstrating their classification performance. In addition, they reveal the models’ validity and predictive ability in terms of the stroke class. Stacking classification outperforms the other methods, with an AUC of 98.9%, F-measure, precision and recall of 97.4% and an accuracy of 98%. Hence, a stacking method is an efficient approach for identifying those at high risk of experiencing a stroke in the long term. The AUC values show that the model has a high predictive ability and distinguishability among the two classes. The future purpose of this study is to enhance the ML framework via the employment of deep learning methods. Finally, a challenging but promising direction is to collect image data from brain CT scans and to evaluate the predictive ability of deep learning models in stroke occurrence.

## Figures and Tables

**Table 1:** Features importance in the balanced data.

Random Forest		Information Gain	
**Attribute**	**Rank**	**Attribute**	**Rank**
Age	0.4702	Age	0.75627
BMI	0.404	Ever_married	0.09382
Avg_glucose_level	0.1139	BMI	0.06991
Ever_married	0.0929	Avg_glucose_level	0.06265
Work_type	0.0898	Work_type	0.05651
Smoking_status	0.0661	Heart_disease	0.02777
Residence_type	0.0537	Smoking_status	0.02554
Gender	0.0500	Residence_type	0.02129
Heart_disease	0.0499	Gender	0.01667
Hypertension	0.0177	Hypertension	0.00523

**Table 2:** Average performance of ML models.

	Precision	Recall	F-Measure	AUC	Accuracy
**NB**	0.812	0.860	0.835	0.867	0.84
**LR**	0.791	0.791	0.791	0.877	0.79
**3-NN**	0.918	0.916	0.915	0.943	0.81
**SGD**	0.791	0.791	0.791	0.791	0.88
**DT(J48)**	0.909	0.909	0.909	0.927	0.91
**MLP**	0.884	0.881	0.881	0.929	0.92
**MVoting**	0.93	0.93	0.93	0.93	0.93
**RF**	0.966	0.966	0.966	0.986	0.97
**Stacking**	0.974	0.974	0.974	0.989	0.98

**Table 3:** Comparison of ML models performance.

	Precision		Recall		F-Measure		Accuracy	
	**Proposed**	**[35]**	**Proposed**	**[35]**	**Proposed**	**[35]**	**Proposed**	**[35]**
**NB**	0.812	0.786	0.860	0.857	0.835	0.823	0.84	0.82
**LR**	0.791	0.775	0.791	0.760	0.791	0.776	0.79	0.78
**3-NN**	0.918	0.774	0.916	0.838	0.915	0.804	0.81	0.80
**DT**	0.909	0.909	0.909	0.775	0.909	0.776	0.88	0.66
**RF**	0.974	0.720	0.974	0.735	0.974	0.727	0.98	0.73

## Data Availability

Not applicable.
